# Ultrasonically enhanced rifampin activity against internalized *Staphylococcus aureus*

**DOI:** 10.3892/etm.2012.758

**Published:** 2012-10-22

**Authors:** SI-FENG SHI, XIAN-LONG ZHANG, CHEN ZHU, DE-SHENG CHEN, YONG-YUAN GUO

**Affiliations:** Department of Orthopaedic Surgery, Shanghai Sixth People’s Hospital, Shanghai Jiao Tong University School of Medicine, Shanghai 200233, P.R. China

**Keywords:** ultrasound, osteoblast, *Staphylococcus aureus*, rifampin

## Abstract

*Staphylococcus aureus* (*S. aureus*) is the principle causative agent of osteomyelitis, accounting for 80% of all human cases. *S. aureus* internalized in osteoblasts escapes immune response, including engulfment by phagocytes. It also escapes the action of a number of antibiotics. Ultrasound increases cell membrane permeability to a number of drugs. Following an internalization assay, we used low-frequency, low-power ultrasound combined with the antibiotic rifampin to target *S. aureus* internalized in human osteoblasts. Tryptic soy agar (TSA) was used to quantitate the antibacterial effect of rifampin combined with low-frequency ultrasound. A Cell Counting Kit-8 (CCK-8) assay was used to evaluate cell viability following exposure to ultrasound. Our data revealed that rifampin successfully penetrates into osteoblasts and kills internalized *S. aureus* in osteoblasts, while low-frequency ultrasound promotes this process. Ultrasound had a negative impact on the cell viability of osteoblasts; however, this damage was slight and reversible. Ultrasound-enhanced antibiotic efficiency to bacteria internalized in the osteoblasts may contribute to the control of chronic infection to reduce recurrence.

## Introduction

Osteomyelitis is an inoculation of bacteria into bone as a result of hematogenous seeding, surgical contamination, spread of infection from an adjacent area, trauma coinciding with contamination or injury to a limb without the protective soft tissue envelope (1). The implantation of foreign bodies increases the likelihood of infection. In this disease, bone is colonized with microorganisms, with associated inflammation and bone destruction (2). Chronic infection is difficult to eradicate. Aggressive surgical debridement of the infected or necrotic tissue and extensive bone and soft tissue reconstruction are usually required to cure the disease (1). Foreign bodies need to be removed in the majority of cases in order to eradicate infection. A small proportion of seriously ill patients require amputation. Besides surgical treatment, intravenous antibiotics for extended periods of time is another form of treatment for osteomyelitis. However, due to the locally compromised blood supply, a therapeutic level of antibiotics is rarely achieved (1,3).

The gram-positive organism *Staphylococcus aureus* (*S. aureus*) is the principle causative agent of osteomyelitis, accounting for 80% of all human cases (4,5). Adhesion molecules of *S. aureus* facilitate its binding to the bone matrix. Toxin secretion such as interleukin (IL)-1, IL-6 and tumor necrosis factor (TNF)-α may produce *S. aureus*-induced osteomyelitis and stimulate bone resorption (6). The bacterial biofilm is considered to be the main reason for refractoriness of osteomyelitis. However, previous research demonstrated that *S. aureus* not only colonized bone matrix, but also invaded osteoblasts (2,5,7–9). This phenomenon has been demonstrated *in vitro* and *in vivo*(10). The capability of *S. aureus* to invade and survive within osteoblasts may be an important reason as to why chronic osteomyelitis is difficult to be eradicate. *S. aureus* internalized in osteoblasts avoids immune responses, including engulfment by phagocytes, as well as the action of many forms of antibiotics. *S. aureus* survived in the intracellular environment of osteoblasts and maintained the vitality to invade other osteoblasts (1,11). This may explain the recurrence and chronic course of this disease (3).

Numerous types of antibiotics have been employed in the treatment of osteomyelitis, including gentamicin, cephalosporins, vancomycin, clindamycin and rifampin. However, the majority of these antibiotics cannot penetrate eukaryotic cells. Rifampin is a hydrophobic antibiotic and is able to penetrate cell membranes and enter osteoblasts. A study *in vitro* demonstrated its bactericidal effect in the intracellular environment (3). In clinical practice, rifampin also demonstrates satisfactory results in the treatment of osteomyelitis, particularly in prosthesis infections (12–15). Other antibiotics capable of penetrating eukaryotic cells, including clindamycin, also have antibacterial effects in the intracellular environment (3).

In order to kill bacteria internalized in the osteoblasts, it is necessary to increase the intracellular concentration of antibiotics. The hydrophobic properties of the drugs and the permeability of cell membranes are two important factors when determining the concentration of a drug in the intracellular environment. The aim of the present study was to improve the permeability of the osteoblast membrane in order to increase the concentration of antibiotics to kill *S. aureus* in the intracellular environment.

The application of ultrasound as an adjunct has showed satisfactory results in the treatment of tumors, infection and DNA transfection in *in vitro* and *in vivo* research. It increases the permeability of eukaryotic and prokaryotic cells to drugs, DNA and nanoparticles. Previous research has documented that ultrasound enhances the activity of a number of antibiotics against certain bacteria in plankton and in biofilms (16,17). Additionally, further research revealed that ultrasound allows antibiotics to transport through the biofilm more easily and therefore increases the drug concentration surrounding the bacteria (18). Ultrasound perturbs the bacteria cell membrane, rendering it more permeable to antibiotics. Ultrasound-enhanced antibiotic efficacy has also been demonstrated in planktonic bacteria. However, no research has reported on the ultrasonic adjuvant effect to bacteria in the intracellular environment. The present study tests the hypothesis that low frequency, low-power ultrasound enhances the antibiotic (rifampin) effect of *S. aureus* internalized in human osteoblasts (19).

## Materials and methods

### Bacterial strains, media and antibiotics

Cultures of *S. aureus* (ATCC12598) were grown on nutrient agar plates. Tryptic soy broth (TSB; Difco, Detroit, MI, USA) was inoculated with one colony from the plate and a culture was grown overnight at 37°C with agitation to the plate.

### Cell culture

Normal human osteoblasts (sv 40) were incubated at 37°C under a humidified atmosphere containing 5% CO_2_ in Dulbecco’s modified Eagle’s medium (DMEM; Gibco, Beijing,China) supplemented with 10% (v/v) fetal bovine serum. Once the cells had reached ∼80% confluence, they were removed from the flasks using 0.025% trypsin and 0.01% ethylenediaminetetraacetic acid (EDTA) in phosphate-buffered saline (PBS). The growth medium was changed every 48 h after seeding. The study was approved by the ethics committee of the Department of Orthopaedics, The Sixth Affiliated People’s Hospital, Shanghai Jiao Tong University School of Medicine, (Shanghai, China).

### Internalization assay

*S. aureus* was grown overnight (16 h) in 5 ml TSB in a water bath at 37°C with agitation. The bacteria were harvested by centrifugation for 5 min at 5,000 rpm at 4°C and washed twice in 5 ml PBS. The pellet was then resuspended in 5 ml osteoblast growing medium (OBGM) without antibiotics. Confluent cell layers of osteoblasts were washed three times with 5 ml PBS to remove growth media containing antibiotics. Osteoblasts were then infected at a multiplicity of infection (MOI) of 200:1 with *S. aureus*. After infection for 45 min at 37°C, cell cultures were washed and then incubated with OBGM containing 100 *μ*g/ml gentamicin for 4 h to kill the remaining extracellular *S. aureus*. Gentamicin cannot penetrate normal eukaryotic cells, so at this time, only intracellular bacteria remain.

### Ultrasound treatment combined with antibiotic activity

Non-focused ultrasonic transducers were used in this experiment to deliver the ultrasound. A function generator (XinZhi, Biotechnology Co., Ningbo, China) created a non-continuous wave at a frequency of 20 kHz. The osteoblasts were divided into 4 groups and treated as follows: in group A, osteoblasts with OBGM containing 30 *μ*g/ml of rifampin were exposed to ultrasound for 60 cycles of 5 sec ultrasound with 2 sec intervals (200 W). In group B, osteoblasts were exposed to rifampin without ultrasound. In group C, the osteoblasts were exposed to ultrasound with OBGM not containing rifampin and rifampin was added to OBGM 30 min after exposure to ultrasound. In group D, the osteoblasts were not exposed to ultrasound.

### Assay to determine rifampin antibiotic activity combined with ultrasound

After rifampin addition and ultrasound intervention (6 h later), osteoblast cultures were washed with PBS 3 times and subsequently lysed by the addition of 1 ml 0.2% Triton X-100 (Fisher Biotech, Fair Lawn, NJ, USA), followed by standard serial dilution, plating on tryptic soy agar (TSA), incubation at 37°C overnight and enumeration of resulting colony forming units.

### Osteoblast viability following ultrasound exposure (CCK-8)

Human osteoblasts (sv40) were inoculated in a 96-well plate. Once the cells had reached ∼80% confluence, they were exposed to ultrasound as previously described. The osteoblasts were then cultured in standard environment for another 24 h. The Cell Counting Kit-8 (CCK-8; Beyotime, Shanghai, China) was employed to quantitatively evaluate osteoblast viability (20). Briefly, the culture medium was removed and the cultures were washed with PBS twice. Serum-free DMEM (∼100 *μ*l) and CCK-8 (10 *μ*l) solution were added to each well. Following incubation at 37°C for 2 h, a microplate reader (BioTek Instruments, Winooski, VT, USA) was employed to determine the optical density (OD) at 450 nm and compared with the control group. Three duplicate experiments were performed to assess the cell viability. The following equation was used: cell viability = (OD_sample_ / OD_control_) × 100.

### Statistical analysis

Each experiment was performed three to six times and the quantitative results were expressed as mean ± standard deviation. Differences between the means were analyzed by using independent t-test. P<0.05 was considered to indicate a statistically significant difference.

## Results

### Internalization of S. aureus

**Figure 1:**
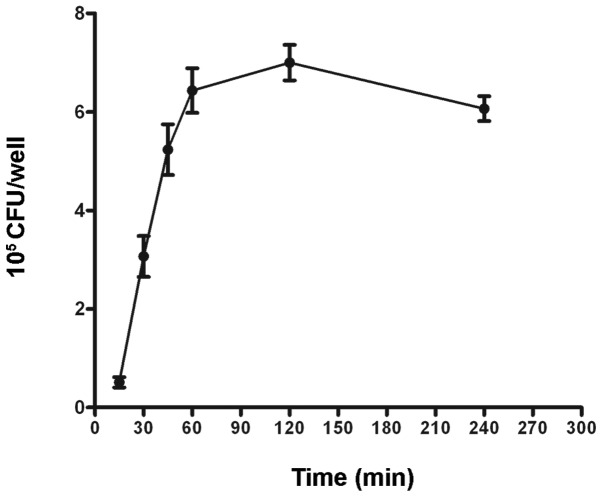
Quantification of internalized *S. aureus* in human osteoblasts. The sv40 human osteoblasts were exposed to *S. aureus* ATCC12598 at an MOI of 200 for the indicated time. Gentamicin was used to kill the remaining extracellular *S. aureus* for 4 h. The osteoblasts were lysed by the addition of 1 ml 0.2% Triton X-100, followed by standard serial dilution, plating on tryptic soy agar, incubation at 37°C overnight and enumeration of resulting colony forming units. CFU, colony forming unit; MOI, multiplicity of infection.

**Figure 2:**
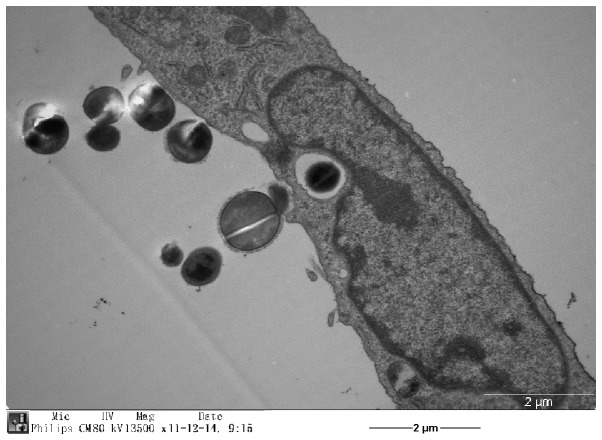
Internalization of *S. aureus* by an osteoblast observed under transmission electron microscopy.

We quantified the invasion of *S. aureus* into osteoblasts using TSA plates. Following an internalization assay, the osteoblast cultures were washed three times and subsequently lysed by the addition of 1.2 ml 0.1% Triton X-100. Suspension dilutions of the lysates were plated in triplicate on TSA plates followed by incubation at 37°C overnight. Results showed that the invasion of *S. aureus* increased in a time-dependent manner following infection at an MOI of 200 (Fig. 1). Internalization of *S. aureus* by osteoblasts was confirmed by transmission electron microscopy (Fig. 2).

### Effect of ultrasound combined with antibiotic inhibition of S. aureus internalized in osteoblasts

**Figure 3:**
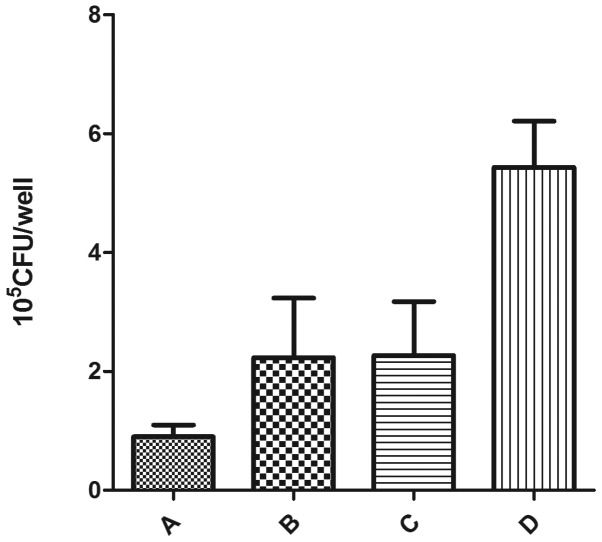
Quantification of internalized *S. aureus* in human osteoblasts following exposure to ultrasound and rifampin. (A) Osteoblasts with OBGM containing 30 mg/ml of rifampin were exposed to ultrasound; (B) osteoblasts were exposed to rifampin without ultrasound; (C) osteoblasts were exposed to ultrasound with OBGM not containing rifampin and rifampin was added to OBGM 30 min after exposure to ultrasound; (D) osteoblasts were not exposed to ultrasound or rifampin. CFU, colony forming unit; OBGM, osteoblast growing medium.

When *S. aureus* cells were exposed to antibiotics combined with ultrasound, the number of viable organisms in the intracellular environment of osteoblasts decreased. Rifampin, a bactericidal nucleic acid synthesis inhibitor, is readily able to penetrate eukaryotic cells. The number of viable *S. aureus* cells within osteoblasts not treated with ultrasound was smaller compared with the control group, but larger compared with that of rifampin combined with ultrasound. There was no significant difference between group B and group C. These results suggest that rifampin successfully penetrates osteoblasts and kills *S. aureus* internalized in the osteoblasts, while ultrasound promotes this process (Fig. 3).

### Osteoblast viability following ultrasound exposure

**Figure 4:**
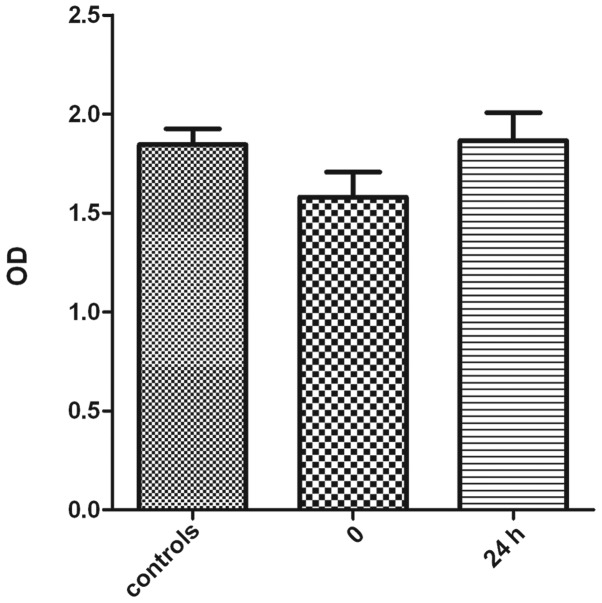
Comparison of osteoblast viability immediately after exposure to ultrasound, 24 h after exposure to ultrasound and the control group without exposure to ultrasound. OD, optical density.

The CCK-8 assay was employed to compare viability of sv40 osteoblasts following exposure to ultrasound with normal cells. The results revealed a decreased viability of the osteoblasts immediately after exposure to ultrasound. However, the viability of osteoblasts exposed to ultrasound increased after being cultured in the standard atmosphere for 24 h. There was no significant difference in viability of osteoblasts 24 h after exposure to ultrasound and the control group (Fig. 4). These results suggest that low-frequency ultrasound has a negative impact on cell viability of osteoblast sv40; however, this damage is slight and reversible.

## Discussion

Ultrasound has been used in a number of applications in medicine and research (16,18,21,22). Previous research has shown that low-frequency ultrasound, when combined with antibiotics, significantly enhances the bactericidal action of antibiotics (16,22,23). Previous research has shown that ultrasound effectively enhances the antibacterial effect of certain antibiotics against a culture of bacteria in *in vitro* and *in vivo* biofilms (17,23). Ultrasound increases the transport of antibiotics through biofilms, which is a barrier for antibiotics to penetrate in order to kill bacteria inside (18). Carmen *et al* demonstrated that ultrasonication significantly increases the transport of gentamicin through the biofilm. Insonation of biofilms for 45 min more than doubles the gentamicin concentration compared to the non-insonated counterparts. This enhanced transport may be partially responsible for the increased killing of biofilm bacteria exposed to combinations of antibiotics and ultrasound (18). Ultrasound-enhanced antibiotic efficacy has also be conducted in *in vivo* research. Carmen *et al* investigated the combination of low frequency ultrasound and vancomycin in treating *Staphylococcus epidermidis* infections in a rabbit model and the result showed enhanced vancomycin activity against *S. epidermidis* biofilms. At 48 h of insonation there were significantly fewer viable bacteria in the insonated biofilm (17).

However, ultrasound-enhanced antibiotic activity in planktonic cultures with no extensive exopolymer matrix to hinder antibiotic transport, indicates that there must be other mechanisms involved in the ultrasound-enhanced antibiotic effect on organisms. Ultrasound is postulated to increase antibiotic concentration inside cells by rendering the cell membrane more permeable to the antibiotic (19). Perturbation caused by ultrasound results in decreased stability of the phospholipid bilayer. Rapoport *et al* observed that the passage of stearic acid (lipophilic substances) into the outer lipid bilayer was ultrasonically enhanced in *Pseudomonas aeruginosa*(24,25).

A similar reversible destabilization may occur in eukaryotic cell membranes. Studies have indicated that low-frequency ultrasound destabilizes lipid layers in the skin, thus enhancing the permeability of drugs (16,26). It has also been demonstrated that plasmid DNA was effectively delivered into *Saccharomyces cerevisiae* by using low-frequency ultrasound (16,27). Our current study indicates that ultrasound may create perturbations in the outer membrane lipid bilayer of osteoblasts sufficiently large for the passage of rifampin.

Recently, research into ultrasound-aided cancer chemotherapy has been conducted to enhance the overall cytotoxic effect of the therapeutic agent with no apparent hyperthermic event (28). Sonoporation is an ultrasound-induced means of increasing the permeability of cell membranes to promote the passage of chemotherapy agents across the cell membrane, thereby facilitating entry of the relevant drug into the target cell (29–31). Our results are consistent with the previous result. Rifampin is a lipophilic drug and the ultrasonically-enhanced bactericidal capacity in the intracellular environment may be due to the increased drug concentration in the intracellular environment (19).

Sonoporation has shown promising prospects (32). Although the mechanism by which ultrasound enhances antibiotic action is not fully known, it may be due to perturbation of the cell membrane or stress responses by the bacteria (22,33). Sonoporation enhances cell membrane permeability allowing transfer of macromolecules. Ultrasound-induced biological effects are commonly considered to be caused by acoustic cavitation, which may collapse and lead to the induction of transient holes in the cell membrane. It has been hypothesized that antibiotics are transferred into cells across the cell membrane via ultrasound-induced pores. This is a transient phenomenon and our results also give indirect evidence of this, as the cell viability decreased immediately after the application of ultrasound, but recovered 24 h later (19). Ultrasound-induced pores have also been confirmed by scanning election microscopy (SEM) observations, although the size distribution could not be defined (32).

Ultrasonic waves pass directly through cells with little absorption or scattering. The pressure oscillations of ultrasound produces gas bubbles ranging in size from approximately 1 to 100 *μ*m in diameter in the liquid. The oscillations of bubbles, also called cavitation, are generally divided into ‘stable’ and ‘collapse’ types. Both types of cavitation are reported to increase membrane permeability in eukaryotic cells (19). Stable cavitation is the low intensity oscillation of the bubbles without complete collapse and collapse cavitation occurs at higher intensity levels.

There is an intensity threshold for the production of collapse cavitation and collapse cavitation is absent below this threshold; however, stable cavitation occurs readily (19,34). The lower shear forces caused by stable cavitation may also be sufficiently stressful to perturb the outer membrane (35). Ultrasound has been shown to enhance fluorescent probe uptake in corneal endothelium and various cancer cells, as well as allow penetration of Ara-C into HL-60 cells and allow transfection of eukaryotes with plasmid DNA (19,28,32,36,37). When these bubbles collapse, they violently accelerate the fluid around them, producing a high temperature and free radicals as well as generating high liquid shear force. Cell membranes may become stressed or damaged by shear force, heat or free radicals (38). Stress and high velocity jet of liquid toward the cell membrane during bubble expansion may contribute to the cell membranes damage (19,39).

Although ultrasound is used as a means to lyse bacteria, the low-frequency and lower intensities of ultrasound did not directly lyse the bacterial cells or permanently damage the outer bacterial membrane (19). Indeed, ultrasound at the intensities used in our research did not directly reduce viability of bacteria. In the present study, group C, with ultrasound in the absence of antibiotics, demonstrated no significant decrease in the number of bacteria (Fig. 3). In fact, in certain situations, low-frequency ultrasound increased bacteria cell growth (38). Pitt *et al* used ultrasound to irradiate bacterial cells (*S. epidermidis*, *P. aeruginosa* and *Escherichia coli* cells) attached to polyethylene surfaces. They found that low frequency ultrasound (70 kHz) of low acoustic intensity increased the growth rate of the cells compared to growth without ultrasound. Ultrasound has also demonstrated the ability to enhance planktonic growth of *S. epidermidis* and other planktonic bacteria. These research results may be due to an increased rate of transport of oxygen and nutrients to the cells and waste products away from the cells by ultrasound (38).

Ultrasound increases the permeability of cells without killing them, to allow drugs to enter the cells (34). A major challenge to the application of sonoporation in drug delivery is to optimize the ultrasound parameters to increase the permeability of cell membranes and to maintain good cell viability (40). Acoustic cavitation induced by ultrasound increases cell permeability and facilitates drug internalization in the cells. However, it may also damage or kill the cells if the pores induced by cavitation are too large such that the cell membrane cannot reseal quickly. For large molecules, this is a more serious problem since formation of larger pores is needed on the cell surface, risking disruption to the cell membrane, loss of vital cytoplasmic compounds and resulting in cell death. The results presented in this study show that a reasonable result can be achieved under the correct ultrasound conditions. The cell viability is as high as 80% compared with the control group immediately after sonoporation. However, this is a transient process, as shown in Fig. 4, since cell viability returns 24 h after ultrasound exposure.

The use of ultrasound to transfect eukaryotes with plasmid DNA indicates that the enhanced plasma membrane permeability is transient and the cell membrane can seal or heal to preserve viability (19,32). Mitragotri *et al* used low-frequency ultrasound to destabilize lipid layers and enhance permeability of drugs in the skin. Following ultrasonic treatment, the original permeability was eventually restored (26). Freeze-fracture electron microscopy has shown that, a few seconds after the electric pulse, the pores began to reseal; 5 sec post-electroporation, the majority of the pores became shallow and smaller, while at 10 sec the pore-like structures on the cell membrane had almost disappeared (32,41). Guzman *et al* reported similar results when cells were exposed to low frequency ultrasound (24 kHz) (37,43,44). Uptake of bovine serum albumin and calcein was completely abolished when the compounds were added 1 and 2 min after ultrasound exposure, respectively (18). The short duration of membrane pore opening implies that, if the drug is to be effectively internalized, it should be close to the membrane when poration occurs (32,42–44). Therefore, application of drugs and the interaction between drugs and cells should be performed prior to sonication.

Chronic orthopedic infection is a serious concern in clinical practice. Ultrasound-enhanced antibiotic efficiency to the bacteria internalized in the osteoblast may contribute to the control of chronic infection and reduce the recurrence. Further research is required to allow for better understanding of these fundamental issues and to fully exploit the potential use of sonoporation in treatment of infections.
